# Matrix-Driven Modulation of Phenolic Profiles from *Euterpe oleracea* and *Oenocarpus bacaba* Using Natural Deep Eutectic Solvents

**DOI:** 10.3390/molecules31101762

**Published:** 2026-05-21

**Authors:** Saulo Victor e Silva, María Celeste Gallia, Cristian Sillagana Verdezoto, Leonardo Bajda, Ana Ferrari, Gabriel Araujo-Silva, Jefferson Romáryo Duarte da Luz, Maria das Graças Almeida, Luisa Quesada Romero

**Affiliations:** 1 Departamento de Ciencias de los Alimento y Nutrición, Universidad de Antofagasta, Avenida Universidad de Antofagasta, Antofagasta 02800, Chile; 2 Institute of Research and Development in Process Engineering, Biotechnology and Alternative Energies (PROBIEN), National Council for Scientific and Technical Research (CONICET), Neuquén 8300, Argentina; celestegallia@gmail.com (M.C.G.); leonardo.bajda@probien.gob.ar (L.B.); aferrari@conicet.gov.ar (A.F.); 3 Master’s Program in Innovation in Biosciences and Bioengineering, Faculty of Engineering, Universidad San Sebastián, Concepción 4081339, Chile; csillaganav@correo.uss.cl; 4 Organic Chemistry and Biochemistry Laboratory, Amapá State University (UEAP), Av. Presidente Vargas, s/n, Centro, Macapá 68900-070, AP, Brazil; gabriel.silva@ueap.edu.br; 5 College of Biological Sciences, Federal University of Amapá (UNIFAP), Rodovia BR 156, Universidade, Oiapoque 68980-000, AP, Brazil; jeffduarte@unifap.br; 6 Multidisciplinary Research Laboratory, Department of Clinical and Toxicological Analyses, Health Sciences Center, Federal University of Rio Grande do Norte, R. Gen. Gustavo Cordeiro de Farias, s/n, Petrópolis, Natal 59012-570, RN, Brazil; mgalmeida84@gmail.com; 7 School of Nutrition and Dietetics, Facult of Rehabilitation Sciences and Quality of Life, Universidad San Sebastián, Valdivia 5110466, Chile

**Keywords:** natural deep eutectic solvents, phenolic extraction, antioxidant capacity, HPLC-DAD, amazonian fruits, solvent selectivity

## Abstract

This study investigated the influence of solvent composition on the extraction efficiency and selectivity of phenolic compounds from the Amazonian fruits açaí (*Euterpe oleracea*) and bacaba (*Oenocarpus bacaba*). Six choline chloride-based natural deep eutectic solvents (NaDES), combined with different hydrogen bond donors (glycerol, 1,2-propanediol, citric acid, lactic acid, oxalic acid, and urea), were compared with acidified methanol. Extracts were evaluated for total phenolic content (TPC), total flavonoid content (TFC), and antioxidant capacity using FRAP, DPPH, and ORAC assays. In açaí, methanol exhibited the highest TPC and reducing capacity, whereas acid-based NaDESs enhanced phenolic recovery in a matrix-dependent manner. In bacaba, choline chloride–citric acid enhanced total phenolic recovery compared to methanol, highlighting matrix-dependent solvent performance. Differences among FRAP, DPPH, and ORAC responses reflected variations in phenolic composition rather than total concentration alone. HPLC-DAD analysis revealed solvent-selective enrichment of anthocyanins, chlorogenic acid, flavan-3-ols, and rutin, particularly with acid-based NaDES formulations. Molecular docking provided complementary mechanistic insight by indicating favorable interactions between major phenolics and polyphenol oxidase. Overall, the results indicate that choline chloride-based NaDESs can function as tunable extraction systems capable of modulating phenolic profiles in a matrix-dependent manner, representing promising alternatives to conventional organic solvents for phenolic recovery from Amazonian fruits.

## 1. Introduction

Amazonian fruits are recognized as valuable sources of phenolic compounds, which contribute substantially to the antioxidant properties of plant-derived foods. Among these, *Euterpe oleracea* (açaí) and *Oenocarpus bacaba* (bacaba) have attracted increasing attention due to their rich phenolic composition and commercial relevance in both local and international markets. These fruits contain phenolic acids, flavonoids, and anthocyanins, particularly cyanidin derivatives, associated with strong antioxidant capacity and chemical stability [1,2,3,4].

The recovery of phenolic compounds from complex plant matrices depends strongly on the extraction method employed. Conventional hydroalcoholic solvents, such as acidified methanol, are widely used due to their efficiency; however, they present environmental and operational limitations. In this context, natural deep eutectic solvents (NaDESs) have emerged as promising alternatives for extracting polar bioactives [5]. Formed by mixtures of hydrogen bond donors and acceptors, NaDESs offer tunable polarity, adjustable hydrogen-bonding networks, and physicochemical properties that can be modulated according to component selection [5,6,7].

Recent studies have demonstrated the effectiveness of NaDESs in recovering anthocyanins and flavonoids from fruit matrices [8,9,10,11,12]. Nevertheless, comparative investigations integrating compositional profiling and antioxidant assessment for Amazonian fruits remain limited. In particular, the influence of NaDES composition on phenolic selectivity and antioxidant behavior in açaí and bacaba requires further systematic evaluation.

Analytical techniques such as HPLC-DAD are essential for detailed phenolic characterization, while in vitro assays including DPPH, FRAP, and ORAC provide complementary information regarding antioxidant mechanisms. Additionally, molecular docking has been applied as an auxiliary tool to explore potential interactions between phenolic compounds and oxidative enzymes, contributing mechanistic insight to compositional findings.

Therefore, the present study aimed to compare the phenolic composition and antioxidant properties of açaí and bacaba extracts obtained using selected NaDES formulations and conventional solvent systems. Spectrophotometric assays and HPLC-DAD were employed for quantitative and qualitative characterization, and selected phenolics were further evaluated through molecular docking to examine potential interactions with polyphenol oxidase. This approach provides a comparative framework for understanding solvent-dependent phenolic extraction in Amazonian fruits.

The selection of natural deep eutectic solvents (NaDESs) in this study was based on the need to explore a chemically diverse set of hydrogen bond donors (HBDs) with distinct physicochemical properties that may influence the extraction of phenolic compounds. Specifically, organic acids (citric acid and oxalic acid) were included due to their high polarity and ability to promote acid–base interactions, which may enhance the solubilization and stabilization of phenolic compounds, particularly anthocyanins. Polyol-based systems (glycerol and 1,2-propanediol) were selected for their strong hydrogen bonding capacity and relatively moderate viscosity, which can favor mass transfer during extraction. In addition, urea was chosen as a representative amide-based HBD due to its capacity to disrupt intermolecular hydrogen bonding networks, while proline was included as an amino acid-based HBD with amphiphilic characteristics that may enable specific interactions with both polar and moderately hydrophobic compounds. This selection strategy was designed to evaluate how different interaction mechanisms, including hydrogen bonding, polarity, acidity, and viscosity, influence both extraction efficiency and selectivity toward phenolic compounds from complex plant matrices.

## 2. Results

### 2.1. Phenolic Content, Flavonoids, and Antioxidant Activity

The extraction efficiency of the NaDES systems was evaluated based on total phenolic content (TPC), total flavonoid content (TFC), and antioxidant capacity measured by FRAP, DPPH, and ORAC assays (Table 1 and Table 2).

In açaí, the methanol extract presented the highest TPC, TFC, and FRAP values (*p* < 0.05 vs. all NaDESs), confirming its strong extraction capacity for phenolic compounds. Among the NaDES formulations, A/ChCl/Oxa showed the highest TPC among eutectic systems, while A/ChCl/Pro demonstrated superior TFC compared to the other NaDES extracts. Although lower than methanol, A/ChCl/Oxa exhibited the highest reducing power among NaDESs in the FRAP assay.

DPPH results are presented descriptively, as the assay was conducted in technical triplicate within a single analytical run. Under these conditions, the values should be interpreted as indicative of relative radical scavenging behavior rather than as absolute measures of antioxidant capacity. Variations observed among NaDES extracts may reflect differences in phenolic composition as well as possible solvent–radical interactions inherent to eutectic systems.

ORAC values showed variability among NaDES systems. However, as ORAC determinations were performed in technical triplicate within a single analytical run, these values are presented for comparative purposes only. Differences observed across extracts may be influenced by phenolic subclass distribution and assay-specific sensitivity, and should not be interpreted as directly proportional to total phenolic content.

For bacaba, the extraction profile differed substantially. B/ChCl/Cit yielded the highest TPC, significantly exceeding methanol (*p* < 0.05), whereas B/ChCl/Pro did not differ significantly from methanol. Methanol remained the most efficient solvent for TFC and exhibited the highest reducing capacity in the FRAP assay (*p* < 0.05 vs. NaDESs).

In the DPPH assay, B/ChCl/Oxa and B/ChCl/Cit presented lower IC_50_ values compared to methanol, indicating strong radical scavenging capacity. ORAC results were particularly elevated for B/ChCl/Gly and B/ChCl/Pro, suggesting enhanced oxygen radical absorbance potential in bacaba extracts obtained with these solvent systems.

In both matrices, the urea-based NaDES (ChCl/Ure) showed moderate to low extraction performance compared to acid-based systems, particularly in terms of FRAP and ORAC responses. This behavior may be associated with the specific interaction mechanisms of urea at the molecular level. Urea is known to disrupt structured hydrogen-bonding networks, which may weaken specific interactions between the solvent system and phenolic hydroxyl groups, thereby limiting solubilization efficiency.

Additionally, the absence of intrinsic acidity in the urea-based NaDES may reduce their ability to stabilize pH-sensitive phenolic compounds, particularly anthocyanins, which are abundant in both matrices. These combined effects may explain the comparatively lower extraction performance observed for ChCl/Ure systems.

The proline-based NaDES (ChCl/Pro) exhibited intermediate extraction performance, with notable variability depending on the matrix and assay. As an amino acid-based HBD, proline presents amphiphilic characteristics that may enable interactions with both polar and moderately hydrophobic compounds. However, its cyclic structure may introduce steric constraints that limit efficient solute–solvent interactions with certain phenolic compounds.

Interestingly, despite moderate TPC values, ChCl/Pro systems showed relatively high ORAC responses in bacaba extracts, suggesting a potential enrichment of specific phenolic subclasses with strong peroxyl radical scavenging capacity. This indicates that proline-based NaDESs may influence extraction selectivity rather than maximizing total phenolic yield.

Overall, the results demonstrate that NaDES performance is both matrix-dependent and solvent-composition-dependent. While methanol showed higher values for total phenolics and reducing power in açaí, acid-based NaDES formulations notably enhanced phenolic extraction and radical scavenging capacity in bacaba.

The phenolic composition of selected extracts was characterized by HPLC-DAD to explore solvent-driven differences in phenolic profiles. For this purpose, the ChCl/Oxa system was selected as a representative acid-based NaDES based on its consistent performance in TPC and FRAP assays across both matrices. This selection does not imply superior extraction efficiency among all tested systems but provides a focused basis for compositional comparison between açaí and bacaba.

The data presented in Table 3 highlight differences in phenolic composition between açaí and bacaba extracts obtained using the same solvent system. These differences reflect matrix-dependent extraction behavior rather than differences in overall extraction efficiency.

In bacaba extracts, B/ChCl/Oxa showed higher total anthocyanin levels compared to the corresponding açaí extract obtained with the same solvent system (46.502 mg/L), along with substantial levels of chlorogenic acid (9.016 mg/L), epicatechin (5.094 mg/L), and rutin (7.753 mg/L). B/ChCl/Lac also showed elevated chlorogenic acid (9.070 mg/L) and rutin (8.760 mg/L) concentrations, highlighting matrix-dependent solvent selectivity.

The higher phenolic concentrations observed in B/ChCl/Oxa compared to A/ChCl/Oxa reinforce the matrix-dependent extraction behavior of acid-based NaDES systems. Bacaba extracts generally displayed a richer anthocyanin and phenolic acid profile when extracted with acid-based formulations.

**Figure 1:**
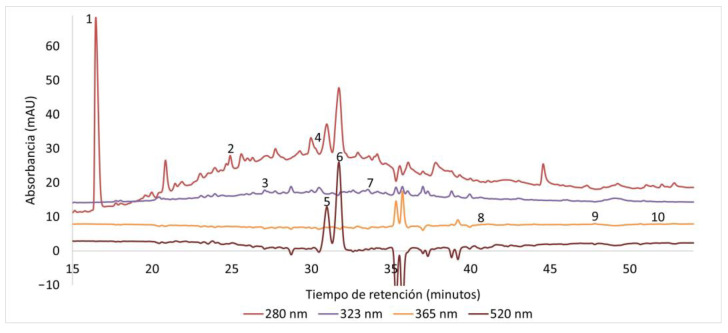
Representative HPLC-DAD chromatogram of açaí (Euterpe oleracea) extract obtained using NaDESs. Peaks correspond to (1) gallic acid, (2) catechin, (3) chlorogenic acid, (4) epicatechin, (5) cyanidin-3-glucoside, (6) anthocyanin derivatives (tentatively identified), (7) ferulic acid, (8) rutin, (9) quercetin, and (10) kaempferol. Quantification was performed only for compounds confirmed with analytical standards. Signal polarity variations are inherent to diode array detection (DAD) and may arise from differences in absorbance between the mobile phase and analytes, as well as matrix-related baseline effects. These variations were interpreted using compound-specific wavelengths and do not affect chromatographic resolution or analytical reliability.

**Figure 2:**
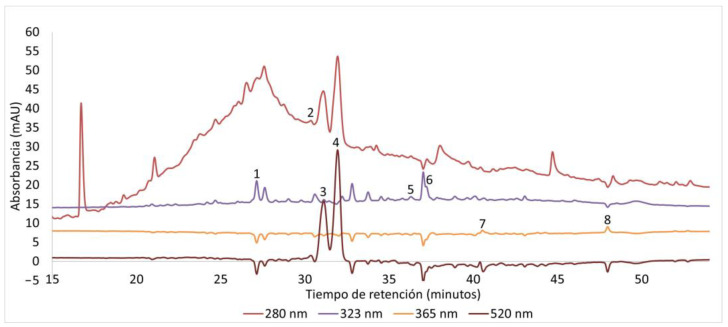
Representative HPLC-DAD chromatogram of bacaba (Oenocarpus bacaba) extract obtained using NaDESs. Peaks correspond to (1) chlorogenic acid, (2) epicatechin, (3) cyanidin-3-glucoside, (4) anthocyanin derivatives (tentatively identified), (5) p-coumaric acid, (6) ferulic acid, (7) rutin, and (8) quercetin. Quantification was performed only for compounds confirmed with analytical standards. Signal polarity variations are inherent to diode array detection (DAD) and may arise from differences in absorbance between the mobile phase and analytes, as well as matrix-related baseline effects. These variations were interpreted using compound-specific wavelengths and do not affect chromatographic resolution or analytical reliability.

Visual inspection of chromatographic profiles (Figure 1 and Figure 2) revealed distinct signal patterns across solvent systems. It is important to note that signal polarity in diode array detection (DAD) may vary due to instrumental and matrix-related effects. In particular, the use of a micro flow cell increases sensitivity to small variations in absorbance and refractive index. Since DAD measures absorbance relative to the mobile phase, signals may appear negative when the mobile phase exhibits higher absorbance than the analytes at a given wavelength, especially at shorter wavelengths.

Additionally, differences between the sample matrix (including NaDES components) and the mobile phase may introduce baseline disturbances, resulting in transient negative deflections. In complex plant matrices such as açaí and bacaba extracts, which are rich in anthocyanins and other phenolic compounds, variability in absorption maxima may further contribute to wavelength-dependent signal inversion.

Importantly, these negative signals do not indicate inadequate chromatographic separation, but rather reflect wavelength-dependent spectral behavior inherent to DAD systems. In our data, negative deflections coincide with positive peaks at the same retention times when monitored at compound-specific wavelengths, as observed for compounds such as chlorogenic acid and cyanidin-3-glucoside (Figure 1 and Figure 2), confirming that these correspond to real analytes.

For this reason, chromatographic interpretation and quantification were performed using compound-specific wavelengths, and only analytically relevant signals were considered. Similar considerations have been reported in HPLC-DAD analyses of phenolic compounds in plant matrices [13,14].

A qualitative association between anthocyanin content and ORAC values was observed; however, this relationship was not uniform across all solvent systems. Given that ORAC determinations were performed descriptively, these trends should be interpreted cautiously.

Overall, HPLC-DAD results demonstrate that NaDES composition strongly influences the selectivity of phenolic extraction. Acid-based eutectic systems favored anthocyanin and phenolic acid recovery, whereas polyol-based systems showed comparatively moderate extraction profiles.

Therefore, HPLC-DAD analysis was not intended to rank extraction efficiency among NaDES systems, but to illustrate how solvent composition modulates phenolic profiles in a matrix-dependent manner.

### 2.2. Molecular Docking Analysis

To provide complementary mechanistic context, selected phenolic compounds identified by HPLC-DAD were evaluated for their interaction with polyphenol oxidase (PPO) through molecular docking simulations. Binding affinities and interaction profiles are summarized in Table 4.

**Figure 3:**
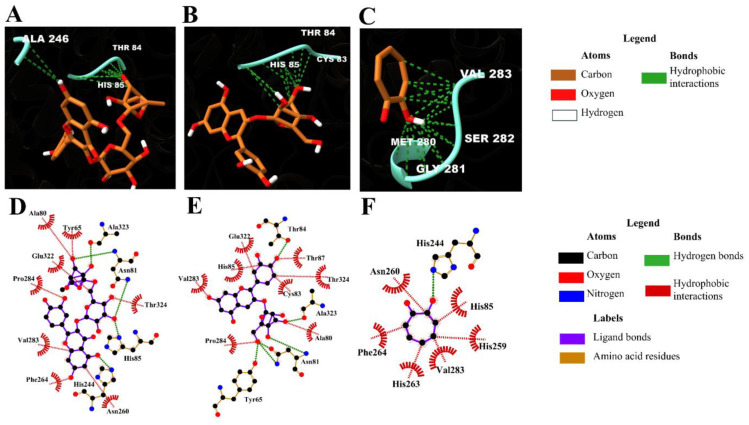
Molecular interactions of rutin (**A**,**D**), cyanidin-3-glucoside (**B**,**E**), and the co-crystallized ligand (**C**,**F**) with residues of the active site of the enzyme polyphenol oxidase. Figures (**A**–**C**) show the three-dimensional structures, while (**D**–**F**) represent two-dimensional diagrams of the same interactions.

**Figure 4:**
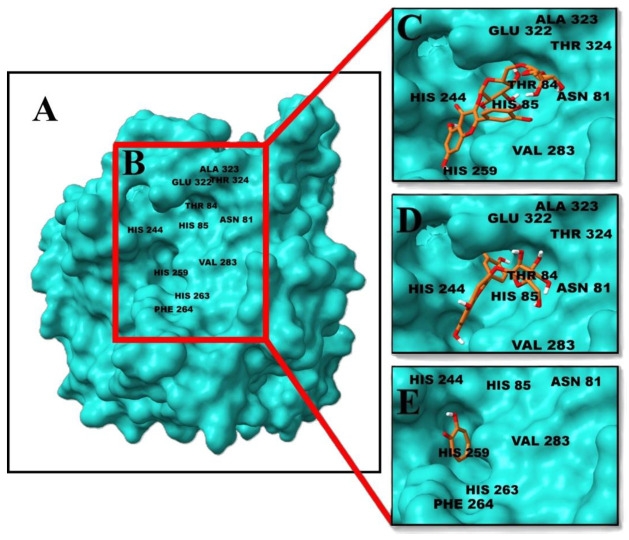
3D visualization of ligand binding positions within the active site of polyphenol oxidase. (**A**) Overall enzyme structure. (**B**) Catalytic site. (**C**) Rutin. (**D**) Cyanidin-3-glucoside. (**E**) Co-crystallized ligand.

As illustrated in Figure 3 and Figure 4, rutin exhibited the highest predicted binding affinity among the evaluated ligands (−7.99 kcal/mol), forming hydrogen bonds with His244, Asn281, and Ala323, together with hydrophobic interactions involving Phe264, Pro284, and Val283 within the catalytic cavity. The ligand was positioned close to the binuclear copper center, indicating a possible interaction with the catalytic region.

Cyanidin-3-glucoside displayed the second highest binding affinity (−6.56 kcal/mol) and established multiple stabilizing interactions within the active site, including hydrogen bonds and hydrophobic contacts with residues surrounding the catalytic core.

Redocking of the native co-crystallized ligand reproduced the binding mode reported by Ismaya et al. (2011) [15], supporting the consistency of the docking protocol and search grid definition.

Molecular docking does not provide direct evidence of biological activity or enzymatic inhibition. In this study, it was used as a complementary approach to explore potential interactions between selected phenolics and the catalytic site of PPO, without establishing functional effects. It is important to note that no consistent linear relationship was observed between total phenolic content and antioxidant assays across all solvent systems. While such correlations are often reported, they are not universal and depend on phenolic composition, structural features, and the reaction mechanism of each assay. In this context, the divergence between TPC, DPPH, and ORAC responses observed in this study likely reflects differences in phenolic selectivity rather than analytical inconsistency.

## 3. Discussion

The present results confirm that solvent composition strongly influences both the efficiency and selectivity of phenolic extraction from açaí and bacaba. Previous studies have demonstrated that choline chloride-based NaDESs can enhance polyphenol solubilization through extensive hydrogen-bond networks and polarity modulation, particularly when combined with organic acids [5,10]. In agreement with these reports, acid-based NaDESs (citric and oxalic acid) showed superior phenolic recovery in bacaba compared to methanol.

In açaí, methanol remained the most efficient solvent for total phenolic extraction, a finding consistent with classical extraction studies reporting the high affinity of hydroalcoholic systems for phenolic compounds [16]. However, A/ChCl/Oxa exhibited comparable reducing capacity in the FRAP assay, suggesting that NaDESs can selectively extract phenolics with high electron-donating potential. Similar observations have been reported for acid-based eutectic systems applied to anthocyanin-rich matrices, where enhanced stability and extraction efficiency were attributed to favorable microenvironmental pH and hydrogen bonding interactions [17,18].

The higher phenolic recovery observed for B/ChCl/Cit and B/ChCl/Oxa in bacaba may be explained by the increased polarity and proton-donating capacity of dicarboxylic acid-based NaDESs. These systems have been shown to improve extraction of anthocyanins and phenolic acids in various plant matrices [5,17], likely due to improved stabilization of flavylium cations under mildly acidic conditions. However, the higher total phenolic content did not directly translate into the highest reducing capacity in all cases, indicating that phenolic subclass distribution and redox-active structural features may exert greater influence on FRAP response than total concentration alone.

Although antioxidant activity is often associated with total phenolic content, the results obtained in this study did not show a consistent linear relationship across all assays. This behavior can be explained by differences in phenolic composition, as well as by the distinct chemical mechanisms underlying each assay. Differences between electron-transfer-based assays (FRAP) and radical scavenging assays with mixed or hydrogen atom transfer mechanisms (DPPH and ORAC) have been widely described. Individual phenolic compounds may respond differently depending on assay chemistry, which can lead to discrepancies when comparing total phenolic content with antioxidant capacity [19,20]. It is important to note that solvent-corrected calibration curves were applied in all colorimetric assays to minimize matrix-related effects associated with NaDES components. Nevertheless, due to the complexity of eutectic systems, interactions with radical-based assays such as DPPH and ORAC may occur. Therefore, these results should be interpreted within a comparative and exploratory framework. Notably, elevated ORAC values were observed for B/ChCl/Gly and B/ChCl/Pro, suggesting that extracts enriched in specific phenolic subclasses may contribute substantially to oxygen radical scavenging capacity.

HPLC-DAD analysis confirmed that acid-based NaDESs enhanced extraction of anthocyanins, chlorogenic acid, and flavan-3-ols. The modulation of phenolic subclasses through solvent selection may therefore influence not only antioxidant metrics but also biological interactions relevant to cardiometabolic and inflammatory conditions [21,22,23]. Comparable selective enrichment of phenolic subclasses using tailored NaDES systems has been reported in recent studies focusing on berry matrices and pigmented fruits [17,24]. These findings reinforce the concept that NaDESs are not merely alternative solvents but tunable extraction systems capable of modulating phenolic profiles. The selective enrichment of specific phenolic subclasses across solvent systems may also be relevant from a pharmacological perspective. Rutin, epicatechin, chlorogenic acid, and anthocyanins, identified as major constituents in selected extracts, have been widely reported for their anti-inflammatory, cardioprotective, and metabolic regulatory properties [25,26,27,28,29]. These biological effects have been associated with modulation of oxidative stress pathways, endothelial function, glucose metabolism, and inflammatory signaling, as documented in recent experimental and clinical investigations [30,31,32,33,34,35]. In this context, matrix-dependent solvent selection appears to influence not only antioxidant outcomes measured in vitro, but also the compositional features that underlie potential bioactivity. While biological assays were not performed in the present study, the observed modulation of phenolic profiles indicates that extraction strategy can shape the functional characteristics of phytochemical-rich preparations. The use of diode array detection allows monitoring at compound-specific wavelengths, improving selectivity in complex matrices such as those extracted with NaDESs.

Docking analysis provides a complementary perspective on the potential interaction of selected phenolics with the catalytic site of polyphenol oxidase. The observed binding affinities and interaction patterns are consistent with previously reported flavonoid–PPO interactions. However, these results should be interpreted as indicative only and do not imply direct biological activity or enzymatic inhibition under experimental conditions. Rutin and cyanidin-3-glucoside demonstrated favorable interactions within the catalytic cavity of polyphenol oxidase, involving residues associated with the binuclear copper center. Similar binding patterns have been reported for flavonoid inhibitors of PPO, suggesting that structural features such as hydroxyl group distribution and glycosylation influence binding affinity [36,37]. In this context, docking results are intended to complement compositional findings rather than to validate antioxidant activity or extraction performance.

Taken together, these results demonstrate that choline chloride-based NaDESs, particularly acid-based formulations, can act as tunable extraction systems influencing the recovery of phenolic compounds from Amazonian fruits. The observed matrix-dependent behavior highlights the importance of solvent selection according to compositional characteristics. Rather than indicating uniformly superior extraction performance, the findings support the role of NaDESs in modulating phenolic profiles. Considering the growing evidence linking phenolic subclasses to specific physiological effects, solvent-driven modulation of extract composition may represent a relevant approach for the design of phytochemical preparations with targeted functional properties [21,30,33].

Although the present study focused on NaDES systems composed of single hydrogen bond donors, the use of mixed NaDESs represents a promising strategy to further enhance extraction performance. The combination of different HBDs may allow the modulation of key physicochemical properties such as polarity, viscosity, and hydrogen bonding capacity, potentially leading to synergistic effects on solute–solvent interactions. In particular, mixed systems could reduce viscosity while maintaining strong solvation ability, thereby improving mass transfer and increasing extraction yields of phenolic compounds.

Therefore, future studies should explore the design of mixed NaDES systems as a means to optimize both total phenolic content (TPC) and total flavonoid content (TFC), as well as to fine-tune selectivity toward specific classes of bioactive compounds.

## 4. Materials and Methods

### 4.1. Chemicals

Rolox (6-hydroxy-2,5,7,8-tetramethylchroman-2-carboxylic acid), fluorescein, 2,2′-azobis(2-amidinopropane) dihydrochloride (AAPH), DPPH, ferric chloride (FeCl_3_), choline chloride, glycerol, 1,2-propanediol, citric acid, lactic acid, oxalic acid, and urea were purchased from Sigma-Aldrich (St. Louis, MO, USA). Methanol, methyl tert-butyl ether (MTBE), and acetonitrile (chromatographic grade) were also obtained from Sigma-Aldrich. Ultrapure water was produced using a Milli-Q purification system (Millipore, Billerica, MA, USA).

Analytical standards used for HPLC analysis were obtained from Sigma-Aldrich. All other analytical-grade reagents and salts were purchased from Synth (São Paulo, Brazil). Prior to chromatographic analysis, all samples and solvents were filtered through 0.22 µm membrane filters (Millipore).

### 4.2. Plant Materials and Sample Preparation

**Figure 5:**
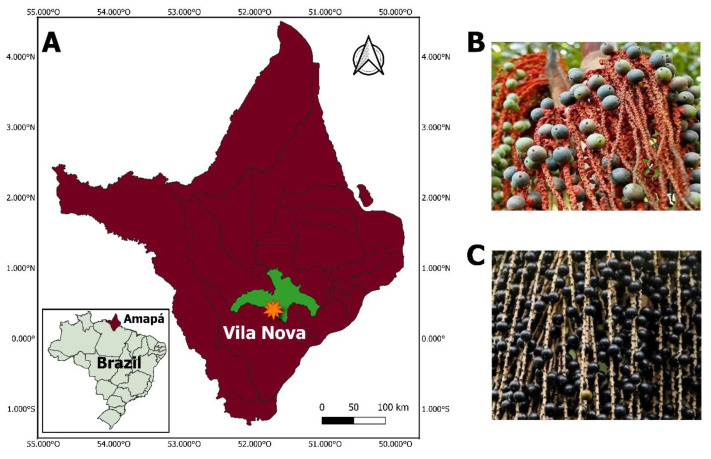
Sample collection area in the community of Vila Nova, Amapá state, Brazil (**A**), *Oenocarpus bacaba* (bacaba fruit) (**B**), *Euterpe oleracea* (açaí fruit) (**C**).

Amazonian fruits (*Euterpe oleracea*—açaí and *Oenocarpus bacaba*—bacaba) were collected between February and March 2024 in the community of Vila Nova, Gaivota (0.408547, −51.735488), located in the state of Amapá, Brazil (Figure 5). After harvest, fruits were washed with distilled water, manually selected to remove damaged material, and sanitized according to standard laboratory procedures at the Organic Chemistry Laboratory of the State University of Amapá.

The edible portion was manually separated to obtain the pulp, which was immediately frozen at −80 °C. The frozen material was subsequently freeze-dried for 72 h. The resulting lyophilized powder was homogenized, passed through a fine mesh sieve, and stored in light-protected containers at room temperature until extraction.

### 4.3. Preparation of Natural Deep Eutectic Solvents (NaDESs)

NaDES formulations were prepared according to El Kantar et al. (2019) [37] by combining choline chloride (hydrogen bond acceptor) with different hydrogen bond donors: glycerol (ChCl/Gly), 1,2-propanediol (ChCl/Pro), citric acid (ChCl/Cit), lactic acid (ChCl/Lac), oxalic acid (ChCl/Oxa), and urea (ChCl/Ure). The molar ratios of each system are presented in Table 5.

The components were weighed according to the specified molar ratios and mixed in sealed glass flasks. Distilled water (30%, *v*/*v* relative to the NaDES base composition) was added during the mixing process to reduce viscosity and facilitate eutectic formation. The mixtures were heated at 80 °C in a water bath under constant stirring for 1 h until a clear and homogeneous liquid was obtained, indicating successful formation of the eutectic system. The final volume of each formulation was adjusted to 10 mL, and the solvents were stored at room temperature in sealed containers until use.

### 4.4. Ultrasound-Assisted Extraction Procedure

Extraction was performed using a two-step ultrasound-assisted procedure. Initially, 10 mL of the extraction mixture containing 100 mg of the freeze-dried sample were processed with a probe sonicator (Intelligent Ultrasonic Processor, model UCD-950; 20–25 kHz; 10–1000 W, BIOBASE, Jinan, China). The probe tip was immersed approximately 2 cm into the sample, and sonication was applied at 40% amplitude for 10 min to promote cell disruption and enhance solvent penetration.

Immediately after probe treatment, samples were transferred to an ultrasonic bath (45 kHz; Biobase, Bioyu Co., Ltd., Jinan, China) and sonicated for an additional 10 min at 30 °C to ensure uniform mass transfer and improved solubilization of phenolic compounds.

Following extraction, samples were centrifuged at 3000 rpm for 25 min (Eppendorf^®^ 5810 R, Hamburg, Germany). The supernatants were filtered through Whatman No. 3 filter paper, collected in amber glass vials, and stored at 4–8 °C until analysis.

All extractions were performed in triplicate for each solvent system.

### 4.5. Determination of Total Phenolic and Flavonoid Contents

Total phenolic content (TPC) was determined using the Folin–Ciocalteu method as described by Jiménez-Aspee et al. (2014) [13], with volume adjustments to adapt the assay to a 96-well microplate format. Gallic acid was used to construct the calibration curve from a 600 µg/mL stock solution prepared in 80% methanol. For the assay, 15 µL of extract solution (8 mg/mL) were mixed with 165 µL of 10% Folin–Ciocalteu reagent and incubated at 37 °C for 5 min. Subsequently, 120 µL of 7% sodium carbonate (Na_2_CO_3_) were added, and the mixture was incubated for 1 h at 37 °C under continuous mixing. Absorbance was measured at 765 nm using a Synergy H1 microplate reader (BioTek, Winooski, VT, USA).

Results were expressed as grams of gallic acid equivalents per 100 g of dried weight (g GAE/100 g DW). The gallic acid calibration curve followed the equation Y = 1.4735x + 0.0141 (R^2^ = 0.9985), where Y represents absorbance and x the gallic acid concentration.

Total flavonoid content (TFC) was determined using the aluminum chloride (AlCl_3_) colorimetric method according to Simirgiotis et al. (2013) [38], with volume adjustments for 96-well microplate analysis. For the assay, 10 µL of 10% AlCl_3_ were mixed with 10 µL of 1 M sodium acetate, 250 µL of distilled water, and 30 µL of extract solution (8 mg/mL). After 30 min of reaction at room temperature, absorbance was measured at 415 nm using the same microplate reader.

Results were expressed as grams of quercetin equivalents per 100 g of dried weight (g QE/100 g DW). The quercetin calibration curve followed the equation Y = 5.5493x + 0.05 (R^2^ = 0.9963).

Each extract was obtained from three independent extractions (*n* = 3), and all spectrophotometric measurements were performed in analytical triplicate.

### 4.6. Antioxidant Capacity Assessment

#### 4.6.1. Ferric Reducing Antioxidant Power (FRAP) Assay

The FRAP assay was conducted following the method of Barrientos et al. (2020) [39], with slight modifications. The FRAP reagent was freshly prepared by mixing 300 mM acetate buffer, 10 mM 2,4,6-tripyridyl-s-triazine (TPTZ) in 40 mM HCl, and 20 mM FeCl_3_ in a 10:1:1 ratio. For the assay, 20 µL of sample was mixed with 150 µL of FRAP reagent and incubated at 37 °C for 5 min. Absorbance was measured at 593 nm using a Synergy H1 microplate reader (BioTek, Winooski, VT, USA).

Each extract was obtained from three independent extractions (*n* = 3), and measurements were performed in analytical triplicate. Absorbance values were blank corrected by subtracting the reagent blank (A_blank = 0.062). A Trolox calibration curve was constructed within the linear range (0.005–0.150 µmol), yielding the equation y = 4.5569x + 0.0362 (R^2^ = 0.9991). Results were expressed as micromoles of Trolox equivalents per gram of dry weight (µmol TE/g DW).

#### 4.6.2. DPPH Radical Scavenging Activity

The DPPH assay was adapted from Simirgiotis et al. (2016) [40] and Larrazábal-Fuentes et al. (2020) [41]. A standard curve was prepared using gallic acid (250 µg/mL in 80% methanol). For the assay, 50 µL of each diluted extract was mixed with 150 µL of a 400 µM DPPH methanolic solution. After incubation at 37 °C for 30 min in the dark, absorbance was measured at 515 nm using a Synergy H1 microplate reader (BioTek, Winooski, VT, USA).

Each sample was analyzed in technical triplicate within a single analytical run (three wells per concentration). Radical scavenging activity was expressed as IC_50_ (µg/mL), defined as the extract concentration required to inhibit 50% of DPPH radicals. IC_50_ values were determined by nonlinear regression analysis (log[inhibitor] vs. normalized response) using GraphPad Prism 8. As the assay was performed in a single independent run, IC_50_ values are presented descriptively without inferential statistical analysis.

#### 4.6.3. Oxygen Radical Absorbance Capacity (ORAC) Assay

The ORAC assay was performed according to Huang et al. (2002) [19], with slight modifications. Fluorescein (1 µM; 80 µg/mL) was used as the fluorescent probe, and 2,2′-azobis(2-amidinopropane) dihydrochloride (AAPH, 250 µM) served as the peroxyl radical generator. Extracts were diluted to final concentrations of 0.5, 1.0, and 2.0 mg/mL in 75 mM phosphate buffer (pH 7.4). A 400 µM Trolox calibration curve was used for quantification.

The assay was carried out in black 96-well plates with opaque lids. Each well received 150 µL of fluorescein and 25 µL of sample, standard, or blank. After a 30 min pre-incubation at 37 °C, 25 µL of AAPH was added to initiate the reaction. Fluorescence was recorded every 2 min for 120 min at excitation/emission wavelengths of 485/528 nm using a Synergy H1 microplate reader (BioTek Instruments, Winooski, VT, USA).

Each sample was analyzed in technical triplicate within a single analytical run (three wells per concentration). ORAC values were calculated from the area under the fluorescence decay curve (AUC) and expressed as micromoles of Trolox equivalents per 100 g of dry weight (µmol TE/100 g DW). As the assay was performed in a single independent run, results are presented descriptively without inferential statistical analysis.

### 4.7. High-Performance Liquid Chromatography (HPLC-DAD) Analysis

All samples were filtered through a 0.22 µm membrane filter prior to injection and analyzed in duplicate. The phenolic profile was determined using an HPLC system (Agilent 1260, Infinity HPLC system equipped with a quaternary pump (Quat Pump VL), autosampler (ALS), thermostatted column compartment (TCC), diode array detector (DAD), and refractive index detector (RID) (Agilent Technologies, Santa Clara, CA, USA)) equipped with a ZORBAX Eclipse XDB-C18 column (4.6 × 250 mm; 5 µm, Agilent Technologies, Santa Clara, CA, USA). The column temperature was maintained at 25 °C, with a flow rate of 0.5 mL/min and an injection volume of 5 µL.

The mobile phase consisted of 1% phosphoric acid in water (solvent A) and methanol (solvent B). The linear gradient elution program was as follows: 0–10 min, 0–15% B; 10–20 min, 15–30% B; 20–30 min, 30–45% B; 30–40 min, 45–60% B; 40–50 min, 60–75% B; and 50–60 min, 75–90% B.

Peak identification was performed by comparing retention times with those of authentic standards analyzed under identical chromatographic conditions. The standards included catechin, epicatechin, myricetin, kaempferol, quercetin, resveratrol, rutin, and the phenolic acids gallic, ellagic, tannic, chlorogenic, syringic, caffeic, p-coumaric, and ferulic acids. Compound identification was performed at Level 1 (confirmed with authentic reference standards) for all quantified analytes. Peaks not confirmed with analytical standards were considered tentatively identified (Level 3), based on retention time and UV–Vis spectral comparison with literature data, and were not included in quantitative analysis. This classification reflects the level of confidence associated with compound assignment in the absence of confirmation by mass spectrometry. Minor variations in retention times were observed between samples, which may be attributed to differences in solvent composition and matrix effects associated with NaDES systems. These variations did not affect compound identification, as confirmed by standard comparison.

All calibration curves showed coefficients of determination (R^2^) greater than 0.999. Data processing was performed using OpenLab ChemStation software (Rev. C.01.10, Agilent Technologies, Santa Clara, CA, USA).

### 4.8. In Silico Molecular Docking

Polyphenol oxidase (EC 1.14.18.1; tyrosinase type) was selected as the molecular target due to its central role in polyphenol oxidation and enzymatic browning processes, as well as the availability of a well-characterized crystal structure (PDB ID: 2Y9W) suitable for modeling interactions with natural antioxidants such as flavonoids and anthocyanins present in the evaluated extracts [15,42].

The catalytic cavity of polyphenol oxidase comprises two relevant regions for ligand recognition. The first corresponds to the catalytic core, defined by residues His244, His259, His263, Phe264, His85, and Val283, which coordinate the binuclear copper center responsible for enzymatic activity. The second region represents a lateral access channel formed by peripheral residues including Ala323, Glu322, Thr324, Asn81, and Thr84, which contribute to ligand stabilization and orientation within the active site.

The protein structure was retrieved from the Protein Data Bank and prepared using AutoDock Tools v1.5.7. Water molecules and heteroatoms were removed, polar hydrogens were added, and Kollman partial charges were assigned. The native co-crystallized ligand was removed prior to docking simulations.

Selected ligands identified in açaí and bacaba extracts were prepared using AutoDock Tools and Avogadro. Hydrogen was added, Gasteiger charges were assigned, and geometries were energy-minimized using the conjugate gradient method.

The docking grid box was centered on the active site at coordinates X = 5.122, Y = 26.421, Z = 97.023, with dimensions of 18.75 × 24.00 × 21.00 Å, based on structural analysis performed in UCSF Chimera v1.9.

Molecular docking simulations were performed using AutoDock Vina v1.2.3 with an exhaustiveness parameter of 8. The conformation presenting the lowest binding free energy (kcal/mol) was selected as the most representative protein–ligand complex.

To validate the docking protocol, a redocking simulation of the native co-crystallized ligand was conducted to reproduce its experimental binding orientation within the catalytic cavity. Protein–ligand interactions were analyzed using UCSF Chimera (version 1.19), University of California, San Francisco, CA, USA) for three-dimensional visualization and LigPlot+ version 2.3 (European Bioinformatics Institute, Cambridge, UK) for identification of hydrogen bonds and hydrophobic interactions.

### 4.9. Statistical Analysis

Results are presented as mean ± standard deviation (SD). Total phenolic content (TPC), total flavonoid content (TFC), and FRAP values were obtained from three independent extractions (*n* = 3 per extract). Statistical comparisons were performed using one-way analysis of variance (ANOVA), followed by Dunnett’s post hoc test to compare each NaDES extract against the methanol control. Statistical significance was established at *p* < 0.05.

DPPH (IC_50_) and ORAC assays were conducted in technical triplicate within a single analytical run (three wells per sample). As these determinations did not involve independent experimental replicates, no inferential statistical analyses were applied to these parameters, and results are presented descriptively.

## 5. Conclusions

This study demonstrates that solvent composition significantly influenced the extraction efficiency and selectivity of phenolic compounds from açaí and bacaba. Acidified methanol showed higher overall extraction performance in açaí, whereas choline chloride-based NaDESs, particularly acid-based formulations, enhanced phenolic recovery and promoted selective enrichment of anthocyanins and phenolic acids in bacaba.

HPLC-DAD analysis revealed solvent-driven differences in flavan-3-ols, phenolic acids, and anthocyanins, while antioxidant assays reflected matrix-dependent variation among extracts. Molecular docking analysis further supported the potential interaction of major phenolics with polyphenol oxidase, providing mechanistic context for oxidation-related behavior.

Together, these findings indicate that solvent selection shapes phenolic composition in a matrix-dependent manner and can be applied to modulate the bioactive profile of phytochemical-rich extracts.

## Figures and Tables

**Table 1:** Antioxidant activity of açaí fruit through extraction with NaDESs compared to methanol.

Extracts	TPC	TFC	FRAP	DPPH	ORAC
	(mg GAE/100 g DW)	(mg QE/100 g DW)	(μmol TE/100 g DW)	(IC_50_, µg/mL)	(µmol TE/100 g DW)
A/ChCl/Gly	3584.81 ± 272.72 *	330.37 ± 31.78 *	489.24 ± 6.45 *	1.30	66.26
A/ChCl/Pro	3539.57 ± 69.11 *	362.41 ± 12.50 *	534.42 ± 34.15 *	78.30	93.77
A/ChCl/Cit	3856.28 ± 514.53 *	268.30 ± 3.47 *	2386.81 ± 263.21 *	1.40	126.12
A/ChCl/Lac	3554.65 ± 94.18 *	284.32 ± 3.47 *	1887.68 ± 133.29 *	1.40	188.30
A/ChCl/Oxa	3946.76 ± 342.58 *	322.36 ± 12.50 *	4075.70 ± 265.28 *	2.90	63.46
A/ChCl/Ure	3599.76 ± 138.22 *	342.39 ± 15.89 *	719.44 ± 232.48 *	0.60	26.57
A/Met/For	9873.69 ± 420.39	2460.77 ± 216.94	5816.05 ± 563.50	5.10	16.59

A/Met/For: methanol/formic acid (98:2, *v*/*v*), used as the control. TPC: total phenolic content (mg gallic acid equivalents, GAE/100 g DW). TFC: total flavonoid content (mg quercetin equivalents, QE/100 g DW). FRAP and ORAC values are expressed as µmol Trolox equivalents (TE)/100 g DW. DPPH results are expressed as IC_50_ (µg/mL). TPC, TFC and FRAP values were obtained from three independent extractions (*n* = 3) and are presented as mean ± standard deviation (SD). Statistical analysis was performed using one-way ANOVA followed by Dunnett’s test, considering methanol/formic acid as the control. * Differences were considered significant at *p* < 0.05. DPPH and ORAC assays were performed in technical triplicate within a single analytical run and are therefore presented descriptively without inferential statistical analysis. These values are intended for exploratory comparison and should be interpreted with caution, particularly considering the absence of independent experimental replicates and the potential influence of NaDES components on radical-based assays.

**Table 2:** Antioxidant activity of bacaba fruit extracts obtained using NaDESs compared to methanol.

Extracts	TPC	TFC	FRAP	DPPH	ORAC
	(mg GAE/100 g DW)	(mg QE/100 g DW)	(μmol TE/100 g DW)	(IC_50_, µg/mL)	(μmol TE/100 g DW)
B/ChCl/Gly	3479.24 ± 316.71 *	338.44 ± 35.20 *	314.97 ± 11.18 *	4.80	1141.53
B/ChCl/Pro	3931.68 ± 509.87	354.40 ± 27.53 *	366.61 ± 28.13 *	IC_50_ not reached	459.87
B/ChCl/Cit	12,422.43 ± 855.25 *	344.39 ± 13.87 *	1827.44 ± 81.73 *	2.40	ND
B/ChCl/Lac	3630.06 ± 94.18 *	466.52 ± 15.12 *	771.08 ± 29.81 *	47.00	23.32
B/ChCl/Oxa	9587.15 ± 1019.74 *	328.37 ± 38.62 *	1595.08 ± 83.24 *	2.00	41.64
B/ChCl/Ure	3645.14 ± 498.37 *	272.31 ± 15.12 *	312.82 ± 35.55 *	5.30	ND
B/Met/For	5530.29 ± 950.48	2446.75 ± 219.42	3329.15 ± 214.16	5.80	186.71

B/Met/For: methanol/formic acid (98:2, *v*/*v*), used as the control. TPC: total phenolic content (mg gallic acid equivalents, GAE/100 g DW). TFC: total flavonoid content (mg quercetin equivalents, QE/100 g DW). FRAP and ORAC values are expressed as µmol Trolox equivalents (TE)/100 g DW. DPPH results are expressed as IC_50_ (µg/mL). TPC, TFC and FRAP values were obtained from three independent extractions (*n* = 3) and are presented as mean ± standard deviation (SD). Statistical analysis was performed using one-way ANOVA followed by Dunnett’s test, considering methanol/formic acid as control. * Differences were considered significant at *p* < 0.05. DPPH and ORAC assays were performed in technical triplicate within a single analytical run and are therefore presented descriptively without inferential statistical analysis. These values are intended for exploratory comparison and should be interpreted with caution, particularly considering the absence of independent experimental replicates and the potential influence of NaDES components on radical-based assays. ND: not detected under the experimental conditions. IC_50_ not reached: 50% inhibition was not achieved within the tested concentration range.

**Table 3:** Comparative phenolic profile of açaí and bacaba extracts obtained using ChCl/Oxa as a representative acid-based NaDES.

Analyte	Concentration (mg/L)		
	A/ChCl/Oxa	B/ChCl/Oxa	Difference (%)
Ferulic acid	1.775	2.019	+13
Rutin	0.820	7.753	+845
Quercetin	0.384	2.965	+672
Chlorogenic acid	1.696	9.016	+431
Epicatechin	3.040	5.094	+6
Cyanidin-3-glucoside	19.657	28.247	+43.70
Total aAnthocyanins *	37.760	46.502	+23.12

Values expressed in milligrams/liter of extract. * Total anthocyanins expressed as cyanidin-3-glucoside equivalents. Quantification was performed using compound-specific wavelengths and calibration curves based on authentic standards.

**Table 4:** Bonding energy and molecular interactions of the three phenolic compounds with the best coupling to polyphenol oxidase, identified in extracts of açaí and bacaba.

Ligand Name	Binding Affinity (kcal/mol)	Amino Acid Residues Involved	Amino Acid Residues Involved	
		Hydrophobic	H-Bond	Hydrophobic
Rutin	−7.99	Thr 84, His 85, Ala 246	His 244, Asn 281, Ala 323	Tyr 65, Asn 260, Phe 264, Ala 280, Val 283, Pro 284, Glu 322, Thr 324
Cyanidin-3-glucoside	−6.56	Cys 83, Thr 84, His 85	Tyr 65, Asn 81, Thr 84, Ala323	Ala 80, Cys 83, His 85, Thr 87, Val 283, Pro 284, Glu 322, Thr 324
Co-crystallized ligand	−5.06	Met 280, Gly 281, Ser 282, Val 283	His 244	His 85, Asn 260, His 263, Phe 264, His 259, Val 283

**Table 5:** Combination of different types of solvents for the preparation of NaDESs.

NaDES	Solvent	Solvent	Proportion	% H_2_O
ChCl/Gly	Choline chloride	Glycerol	1:2	30
ChCl/Pro	Choline chloride	1,2-propanediol	1:2	30
ChCl/Cit	Choline chloride	Citric acid	1:2	30
ChCl/Lac	Choline chloride	Lactic acid	2:1	30
ChCl/Oxa	Choline chloride	Oxalic acid	2:1	30
ChCl/Ure	Choline chloride	Urea	1:2	30
Met/For	Methanol	Formic acid	98:2	-

## Data Availability

No new data were created or analyzed in this study. Data sharing is not applicable to this article.
